# Supplementary material for: ABCFold: easier running and comparison of AlphaFold 3, Boltz-1, and Chai-1
Source: Bioinform Adv. 2025 Jun 27;5(1):vbaf153. doi: 10.1093/bioadv/vbaf153 (PMC12287924; doi:10.1093/bioadv/vbaf153)
Supplement: vbaf153_Supplementary_Data [file vbaf153_supplementary_data.zip › Supplementary Table 1.docx]

Supplementary Table 1) A comparison of ABCFold, PyMOLfold and ChimeraX. Each method supports multiple modelling methods and represents the results to the user. ABCFold uniquely provides users with a tabulated report containing statistics on the models created.

|  | ABCFold | PyMOLfold | ChimeraX |
| --- | --- | --- | --- |
| AlphaFold3 | ✔ | ✘ | ✘ |
| Boltz-1 | ✔ | ✔ | ✔ |
| Chai-1 | ✔ | ✔ | ✘ |
| ColabFold | ✘ | ✘ | ✔ |
| Protenix | ✘ | ✔ | ✘ |
| PAE plots | ✔ | ✘ | ✔ |
| pLDDT plots | ✔ | ✘ | ✘ |
| Tabulated scores | ✔ | ✘ | ✘ |
